# Laboratory services in the context of prevention of mother-to-child transmission of HIV testing requirements in Copperbelt Province, Zambia: a qualitative inquiry

**DOI:** 10.1186/s12913-023-09747-3

**Published:** 2023-07-14

**Authors:** Jonathan Mwanza, Tanya Doherty, Mwansa Ketty Lubeya, Glenda E. Gray, Wilbroad Mutale, Mary Kawonga

**Affiliations:** 1 School of Public Health, Faculty of Health Sciences, University of the Witwatersrand, Johannesburg, South Africa; 2 Health Systems Research Unit, South African Medical Research Council, Cape Town, South Africa; 3 Department of Obstetrics and Gynaecology, School of Medicine, University of Zambia, Lusaka, Zambia; 4 Office of the President, South Africa Medical Research Council, Cape Town, South Africa; 5 School of Public Health, University of Zambia, Lusaka, Zambia; 6 Department of Community Health, Charlotte Maxeke Johannesburg Academic Hospital, Johannesburg, South Africa

**Keywords:** Sample referral system, PMTCT, Turnaround time, Health system, Laboratory and diagnostic

## Abstract

**Introduction:**

Reliable and timely laboratory results are crucial for monitoring the Prevention of the Mother-to-Child Transmission (PMTCT) cascade, particularly to enable early HIV diagnosis and early intervention. We sought to explore whether and how laboratory services have been prepared to absorb new testing requirements following PMTCT Test-and-Treat policy changes in three districts of Zambia.

**Method:**

We employed in-depth interviews and thematic data analysis, informed by the health system dynamic framework. Twenty-Six health workers were purposively selected and a document review of laboratory services in the context of PMTCT was undertaken. All face-to-face interviews were conducted in three local government areas in the Copperbelt Province (one urban and two rural) between February 2019 and July 2020. We extracted notes and markings from the transcripts for coding. Different codes were sorted into potential themes and the data extracted were put within the identified themes. Trustworthiness was confirmed by keeping records of all data field notes, transcripts, and reflexive journals.

**Results:**

The findings revealed that the health system inputs (infrastructure and supplies, human resources, knowledge, and information and finance) and service delivery were unequal between the rural and urban sites, and this affected the ability of health facilities to apply the new testing requirements, especially, in the rural-based health facilities. The major barriers identified include gaps in the capacity of the existing laboratory system to perform crucial PMTCT clinical and surveillance functions in a coordinated manner and insufficient skilled human resources to absorb the increased testing demands. The centralized laboratory system for HIV testing of mothers and exposed neonates meant facilities had to send specimens to other facilities and districts which resulted in high turnaround time and hence delayed HIV diagnosis.

**Conclusion:**

New guidelines implemented without sufficient capacitation of health system laboratory capacity severely limited the effectiveness of PMTCT program implementation. This study documented the areas relating to health system inputs and laboratory service delivery where greater support to enable the absorption of the new testing requirements is needed.

**Supplementary Information:**

The online version contains supplementary material available at 10.1186/s12913-023-09747-3.

## Background

Globally, 1.4 million pregnant women living with HIV are receiving ART, and the Mother-to-Child Transmission (MTCT) rate is 13% making it a significant contributor to the HIV pandemic, accounting for 9% of new infections globally [1]. In Zambia, the HIV epidemic is generalized, with a national prevalence of about 11.1% in adults 15–49 years, and the HIV prevalence is higher among women than men – 14.2% versus 7.5% [2]. The preliminary findings from the prevention of mother-to-child transmission (PMTCT) evaluation study conducted in Zambia between 2014 and 2016 showed an overall cumulative 9-month MTCT rate of 2.59%. The majority of new HIV infections among children occur through MTCT [3]. As a result, the target set for the elimination of new pediatric HIV infections due to MTCT is a case rate of 50/100,000 live births or less and an MTCT of HIV rate of 5% or less by 2023 [4].

Laboratory services are a critical step in the PMTCT of HIV cascade (diagnosis, linkage to care, engagement in care, retention in care, initiation of antiretroviral therapy, and viral suppression) [5] and Zambia over the years has been revising PMTCT testing requirements (Table 1) following policy changes [6].

**Table 1:** Testing requirements in the PMTCT program of Zambia

Specific Population	When to test	Type of test
Pregnant women, breastfeeding women (and their sexual partners)	During antenatal care (ANC): at first ANC visit and every 3 months if negative In labour and delivery (L&D): test if the last test > 6 weeks ago During postnatal care (PNC): test at first contact if unknown status. Serological test at 6 weeks if negative If breastfeeding: retest every 3 months if negative until the cessation of breastfeeding Partner testing: same time points	Serological HIV test
0 to < 10 years old	At birth, 6 weeks of life, or at first contact	NAT or DBS (which should be sent for HIV DNA PCR) 12–18 months (serological test)
	6 weeks, 6 months,9 months, 12 months and 18 months old	
	24 months old	Serological test

MOH GRZ, “Guidelines for Treatment and Prevention of HIV Infection,” 2020

*NAT* Nucleic Acid Test, *DNA* Deoxyribonucleic Acid, *PCR* Polymerase Chain Reaction

In Zambia, laboratory services are offered within the public health sector by the Ministry of Health and form an essential part of the health system; as diagnostic testing is critical for identifying specific diseases, guiding treatment, determining drug resistance, and supporting disease surveillance [7, 8]. This service is delivered through the provincial referral network of laboratories ranging from higher tier (high throughput) laboratories to lower tier (primary healthcare facilities) offering laboratory or no laboratory services dotted across the province. Laboratory services are mainly offered using a tiered laboratory service approach [6, 7].

Presently, the Ministry of Health (MOH), and it's cooperating partners have expanded the laboratory system and developed various blood-based HIV test strategies for diverse sub-populations, and installed polymerase chain reaction (PCR) testing machines in three tertiary hospitals, each servicing the province and other neighboring provinces or regions [6, 8, 9]. The MOH in the province also employs a “hub and spoke” approach whereby facilities offering basic laboratory services receive samples from multiple source laboratories [10].

Despite these strategies, there has never been a study in Zambia that examined the laboratory system in the context of PMTCT of HIV testing requirements. Therefore, understanding that laboratory services are a sub-system of the overall health system, this study, used the health system dynamic framework [11, 12] to explore the barriers and strategies, if any, which have been applied to capacitate the laboratory services in Zambia to absorb the revised PMTCT testing requirements. This framework was selected as it enabled us to explore health system inputs essential for laboratory services (infrastructure and supplies, human resources, knowledge and information and Finance) as well as service delivery aspects of the laboratory services.

## Method

### Study setting and design

This qualitative study utilized document review and in-depth interviews with health workers from three districts, rural (Lufwanyama) and urban (Kitwe and Ndola), out of the 10 districts in the Copperbelt province, and three main referral hospitals Kitwe Central Hospital, Arthur Davison Children Hospital, and Ndola Central Hospital.

### Selection of study participants

We performed the study in twenty-six purposively selected primary healthcare facilities (six in each of the three districts), district health offices in Lufwanyama, Ndola, Kitwe, and the three main referral hospitals in the Copperbelt Province. Primary healthcare facilities where patients requiring laboratory services are typically seen before being referred to referral health facilities/hospitals were purposively selected for their characteristics (availability of the basic laboratory services and access to these services). The three tertiary hospitals were selected because they were the only facilities at the time of the research offering PCR services in the entire province.

We used snowballing sampling [13] where participants were identified through referrals from health facility in-charges and purposive sampling of interviewees who were health workers familiar with the topic and involved in the PMTCT program. The interviewees had experience with and were involved in the PMTCT program planning and decision-making in their respective health facilities and institutions. Health workers were recruited into the study only if they had work experience in the public sector (Ministry of Health) for five years or more and consented to participate (Table 2).

**Table 2:** Interview participants

Health Worker Type	Primary Health Care Facility	Tertiary	District	TOTAL
Laboratory Personnel	4	3	3	10
Nurse (Maternal & Child Health Department)	14			14
Program Officer- Cooperating Partner			2	2
TOTAL	18	3	5	26

## Data collection

### Document review

The first step in data collection involved the review of documents to map the laboratory system in the province and to achieve this, we reviewed relevant policy documents and the literature on laboratory services in the context of PMTCT. These documents were identified through the MOH website and included technical briefs/reports, published articles and strategic documents (Table 3).

**Table 3:** Documents reviewed

Document/Report	Year/Period	Relevance to the study
1. B. E. Nichols et al., “Impact of a borderless sample transport network for scaling up viral load monitoring: results of a geospatial optimization model for Zambia,” J. Int. AIDS Soc., vol. 21, no. 12, 2018, https://doi.org/10.1002/jia2.25206	2018	Provides insights into the sample network for VL in Zambia
2. C. Kankasa, J,., Simbaya, K., Musokotwane, M.C.,Nambao, E.,Yam, T.,Moyo, L.,Phiri, S.,Kalibala, & A., Moonga, (2018). Evaluation of the prevention of mother-to-child transmission of HIV program in Zambia,1(1),48. Unpublished manuscript	2016	Provided insights into the eMTCT program in Zambia
3. K. Nichols, “Viral Load Specimen Referral Network Report Zambia,” no. November 2016, [Online]. Available: https://aidsfree.usaid.gov/sites/default/files/2017.5.16_vl-eid-ref_zambia.pdf	2016	The document provides insights into the national VL specimen referral network
4. Ministry of Health-MOH GRZ, “Guidelines for Treatment and Prevention of HIV Infection,” 2020	2020	The document provided an understanding of the PMTCT testing requirements (Table 1)
5. Ministry of Health GRZ, “National biomedical laboratory strategic plan 2018—2022,” pp. 1–57, 2018, [Online]. Available: https://www.moh.gov.zm/?wpfb_dl=113. Accessed July 10, 2019	2018	It gives the MOH strategic priorities and direction in the biomedical laboratory

### Individual interviews

The PI (Principal Investigator) arranged appointments and interviewed individual health workers using in-depth interview guides. Interviews were done during their free time to avoid disruption of service provision. We used in-depth interviews because they offer more time for a detailed understanding of a complex issue and enable researchers to get an insight into the phenomenon or experience of interest from the participant's perspective [10, 13, 14]. The interviews allowed us to gain insights from the people who were more conversant about the topic [15].

The study instruments were piloted locally using purposive sampling of primary health facilities in Luanshya District. The district in the pilot study was not included in the main study. Their feedback was used to modify and adapt the main instruments as appropriate. The broad questions that guided the interviews were: (1) Please describe the laboratory and diagnostic services offered in the district health facilities concerning PMTCT guideline changes; (2) Can you tell me more about the laboratory system for PMTCT services within the district from the point of sample collection to referring the samples to a tertiary hospital*.*

After each broad question, we probed further to achieve a deeper understanding of the laboratory system offered in the districts under study. JM, together with research assistants, conducted all the interviews. All interviews were conducted in English, audio-recorded and lasted for thirty to forty-five minutes. All IDIs were conducted within a health facility.

The Principal Investigator was a Doctor of Philosophy (PhD) student who had training in qualitative research methods. There was no prior relationship between the researcher and the study participants, and the PI introduced himself as a Doctor of Philosophy (PhD) student researching as part of his academic requirements. Before data collection, he and the research assistants informed the interviewees of the objectives of the study to promote the participants' understanding of the subject. This was done to establish a good starting point for the interviews.

Data collection continued until saturation of ideas was reached, which was noted when no new ideas were expressed. Data saturation was reached when after preliminary analysis we realized that there was no more new information coming up and after which we conducted one interview to ascertain the saturation of ideas.

In the course of data collection, the PI and research assistants met and discussed the key outcomes to ascertain the saturation of ideas. All the transcripts were simultaneously transcribed and translated verbatim into English. We used direct quotes from the transcribed and translated data from the audio-recorded interviews to clarify the responses from the participants and to ascertain the validity of the data [16]. At the end of each interview, the PI and two research assistants summarized the information provided by the participants to achieve credibility of our finding [13, 15].

Interviews for validation were conducted in November and December 2020 in selected study settings. We asked participants two main protocol questions according to the same questions as the primary interview. Finally, we showed the populated health system dynamic framework (Table 4) and asked if they had any responses that deviated from the table.

**Table 4:** Gives a summary of the main themes, codes and sub-codes that were identified within the elements of the HSD framework. These findings are shown in Fig. 1 with the health system dynamic framework

**Elements of the Health System Dynamics Framework**	**Codes**	**Sub-codes**	
		**Barriers to absorption of PMTCT testing requirements**	**Existing strategies to support absorption**
**Resources (health system inputs)**	Infrastructure & Supply	• Inadequate functional laboratory infrastructure • Supply chain failures	
	Human Resource	• Insufficient laboratory personnel • Inadequately trained personnel • Low knowledge of new PMTCT testing requirements	• More staff by donors • Job aids to guide practice
	Knowledge and Information	• Largely paper-based records system	• Electronic laboratory records system – by donors
	Finance	• Lack of cooperating partners in rural districts to complement government financial support	
**Service Delivery**	Specimen collection and transportation:	• Undefined laboratory network • Unreliable cold chain • Undefined transportation system	• “Hub and spoke” • Expanded cold chain in selected PHCs in urban areas
	Specimen processing and Transmitting of results:	• Centralized PCR service points • Designated dates • Lab-to-lab TAT • Poor inter and intra-laboratory sample referral processes	• Work shifts • Courier motorbike or vehicle

**Fig. 1:**
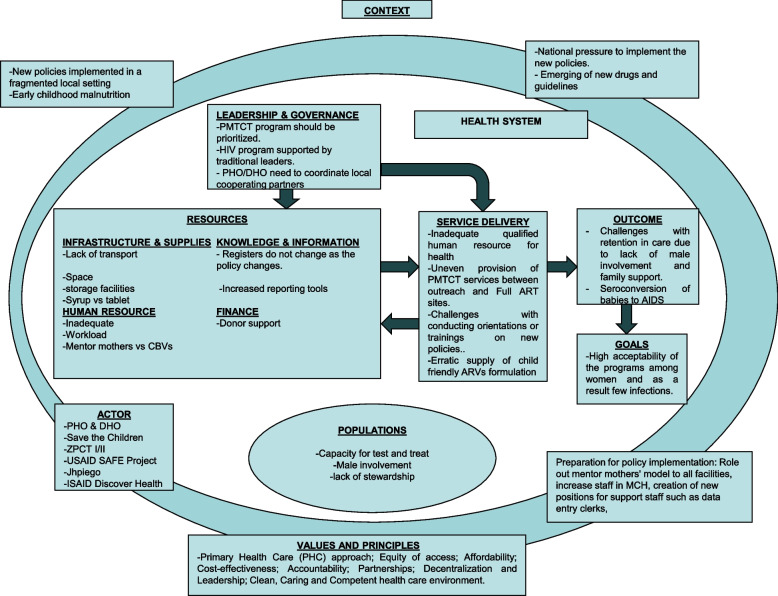
The health system dynamics framework adapted from Van Olmen is populated with the findings from this study

### Data analysis

Data were analyzed thematically according to the Van Olmen health system dynamic framework [10, 14]. This strengthened our understanding of interactions and dynamic relationships between health system inputs and service delivery and the context in which they are implemented. Our multicultural, team-based approach to data analysis, including the involvement of two co-authors who are familiar with the Zambian health system (MK and WM) and two that are not (TD and GG), was used to enhance the transferability of the findings while maintaining credibility [17].

A transcript/memo pair was developed by one researcher, and then reviewed, discussed, and revised in consensus-building meetings with the other analyst to confirm the completeness and rigour of the memos. The memo-writing process identified recurrent ideas as the data manifest to match the objectives of the study. The codes (Table 4) were uploaded to Nvivo 12 Pro software and coded using the identified themes. After coding, code-level queries were run, and analytic memos were written to synthesize the content, make comparisons across participant characteristics, and draw deeper meaning on each theme. The original transcripts were re-visited as needed throughout the coding process to ensure emerging themes were represented and to further contextualize the findings.

Finally, all the documents reviewed were read for familiarization and derived the number of laboratories and services in the province and structure to map the laboratory system in the Copperbelt Province. Special attention was given to the content of the documents describing PMTCT specimen referral networks. This study followed the consolidated COREQ criteria for reporting qualitative research in health care [18].

## Results

### Socio-demographic characteristics of the participants

There were twenty-six health workers aged 30–57 years, including 14 midwives (all females); ten laboratory personnel (4 females, 6 males), and two program Officers (both female).

The results section has been structured to present findings from the document review first (description of the laboratory system and network) then findings from the key informant interviews using sub-headings to align with the main codes in Table 4.

### Health system and laboratory network

**Fig. 2:**
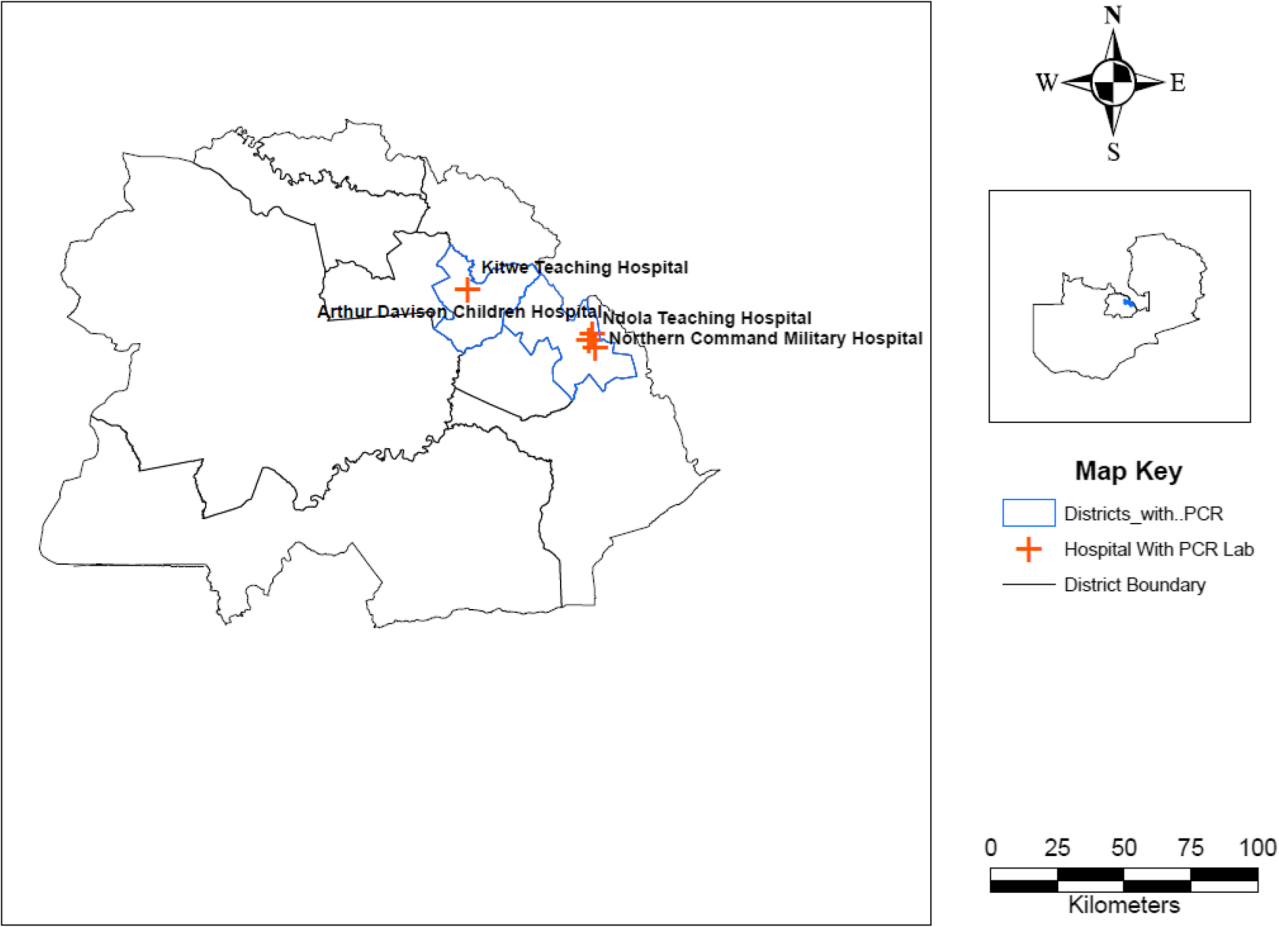
Distribution of Polymerase Chain Reaction (PCR) machines in the Copperbelt Province

The document review demonstrated that the laboratory system in the province begins with health posts, which are usually manned by one or two health personnel. Health posts are usually attached to a health facility that is above this tier. Health facilities with laboratory infrastructure are expected to provide essential laboratory services such as HIV, TB, Malaria diagnostics and other rapid tests, whereas district hospitals and some larger health centres have laboratories staffed with trained technologists who can perform more complex analyses, such as clinical chemistry, haematology, CD4 count, tuberculosis (TB) microscopy, etc. While the majority of hospitals have laboratories attached to them, this is not true for health centres, as only a small number have laboratories. The situation is worse for rural districts as shown in Fig. 2**.** Above this tier, tertiary hospitals have high specimen loads [19] that are facilitated by using more sophisticated, high throughput analyzers in three tertiary hospitals found in Ndola and Kitwe out of the 10 districts servicing other districts and nearby provinces. In Copperbelt Province, samples are collected on specific days from primary health care facilities which are the first contact with clients through a network of couriers and transported to the local source laboratory using the hub and spoke approach. The hub and spoke approach refers to selected higher-tier facilities servicing surrounding low-tier facilities every week to perform a basic repertoire of tests [10]. These latter facilities also act as storage facilities for specialized tests referred to the nearest testing laboratory with an expanded test repertoire such as VL and DBS which is done in centralized tertiary hospitals.

**Fig. 3:**
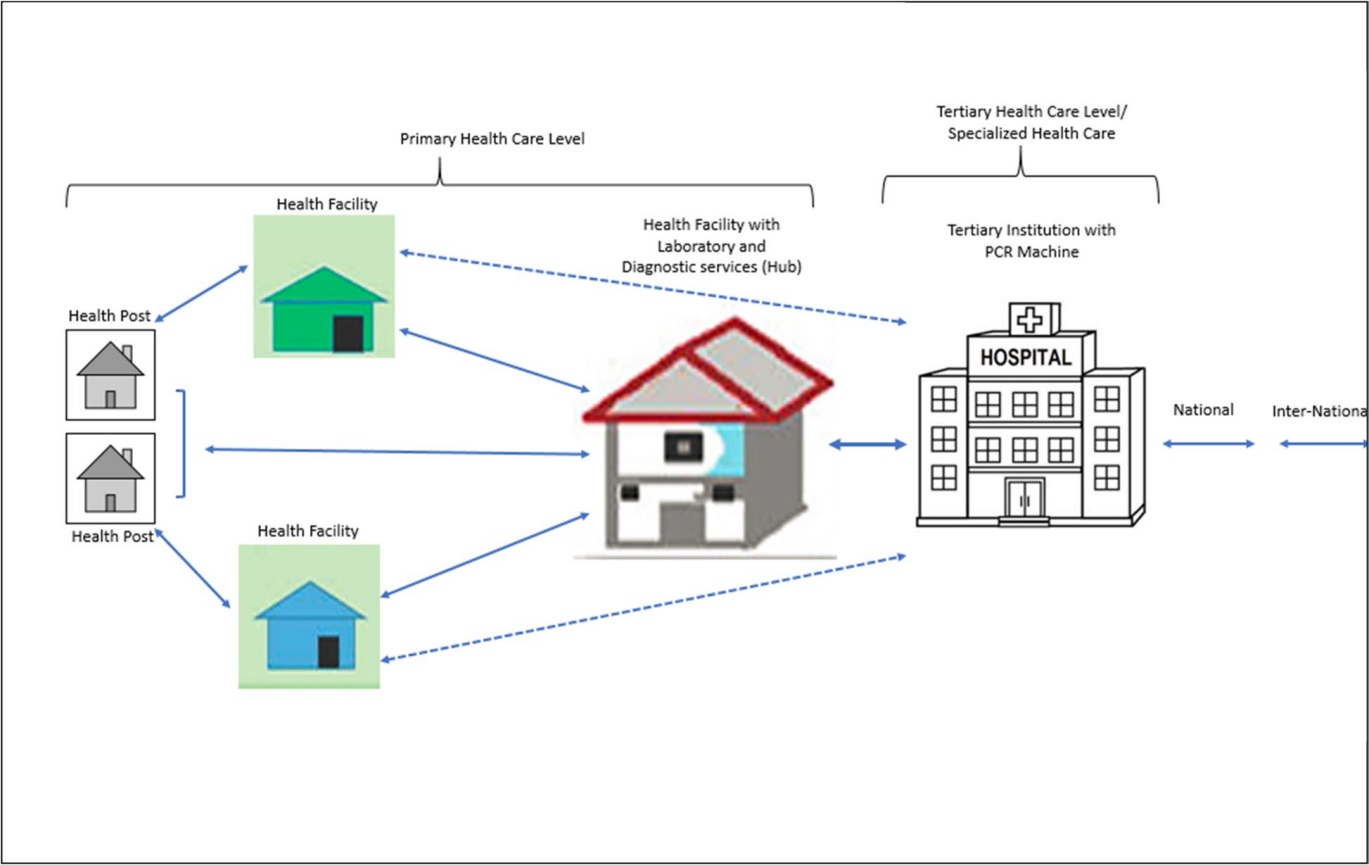
Referral pathways for VL and DBS samples in the Copperbelt Province

The movement of specimens and results takes much longer in rural districts following this process and results are sent back using the same channels used for specimens as shown in Fig. 3**.** The dotted lines (shorter route) show the intermittent route of the samples and results when using the eLab system, a mobile workflow solution that electronically registers VL or DBS samples at the facility and tracks the samples from the facility to delivery and registration at the testing laboratory using a vehicle or motorbike and delivers an electronic result back to the facility with full integration with the in-country laboratory information system [20]. The straight lines (longer route) show the main route of the samples and results when using the paper-based system.

### Resource barriers to absorption of PMTCT testing requirements (health system inputs)

#### Infrastructure and supply

Participants described varying experiences regarding the barriers to the provision of laboratory services for PMTCT in the province. These barriers were identified as inadequate laboratory infrastructure, supply chain failures, and unequal distribution of medical equipment between urban and rural districts. The unequal distribution of medical equipment was also revealed in the document review, as shown in Table 5**.**

**Table 5:** Availability of laboratory infrastructure and services in the study sites

District	Lufwanyama (rural)	Kitwe (urban)	Ndola (urban)
***District laboratory infrastructure available***			
**District Population**	**105,156**	**762,974**	**585,974**
**Number of PHCs (inclusive health posts)**	**32**	**43**	**39**
**PHCs without Laboratory No. (%)**	**26 (81)**	**22 (51)**	**22 (56)**
**PHCs with Laboratory No. (%)**	**6 (19)**	**21 (49)**	**17(44)**
**Tertiary institutions with Polymerase Chain Reaction on HIV Machines**	**0**	**1**	**3**
***PMTCT test requirements: Primary health care facilities laboratory services available***			
**Chemistry tests**			
Liver function tests Kidney function tests	Yes Yes	Yes Yes	Yes Yes, except 1 test
**HIV tests**			
HIV Rapid Tests PCR for HIV DNA sample for referral	No Yes	Yes Yes	Yes Yes
**Haematology tests**			
Full blood count	Yes	Yes	Yes
***PMTCT test requirements: Tertiary health facilities and laboratory services available***			
PCR for HIV DNA	No tertiary facility	KTH: Yes	NTH: Yes ADCH: Yes NCMH: Yes
Early infant diagnosis (EID)	No tertiary facility	NTH: Yes	NTH: Yes ADCH: Yes NCMH: Yes
***Distance to the nearest Laboratory with a PCR Machine***	**102 km** partly by gravel and tarmac road (nearest is KTH)	Chavuma Clinic to KTH: **22 km** representing the longest distance Most facilities within **5 Kms** of the PCR lab PCR Lab serves the whole Province & neighbouring Provinces	NTH to ADCH: **5 km** NTH to NCMH: **4 km** NCMH to ADCH **7 km** PCR Lab serves the whole Province & neighbouring Provinces

*NB* Populations are based on the population and demographic projections for 2011–2035 [21]

Participants cited health facilities in high HIV-burden locations might struggle with the increased patient burden. As a result, the implementation of the Universal Test and Treat policy was a concern due to health system capacity barriers such as poor availability of infrastructure, laboratory reagents and supplies, which may limit same-day ART initiation for individuals newly diagnosed with HIV.

Participants believed that the current Hub and Spoke approach and centralized PCR service points were causing a delay in receiving Dry Blood Spot (DBS), Viral Load (VL) and other testing results, especially for the rural areas which were very far from the centralized service points. As shown in Fig. 2.

#### Laboratory human resources

As identified by participants, insufficient and overburdened health professionals, poor training and lack of knowledge on PMTCT testing requirements were all considered barriers to the absorption of PMTCT policies. As mentioned by one laboratory staff, the changes in the PMTCT cascade increased the workload in the laboratory as a result the staff kept changing their work schedule to meet the demand for more in-coming tests and human resource was needed to process the specimens.

Some respondents indicated that they are unable to attend onsite training due to the workload in the laboratory. Two respondents explained.

#### Knowledge and information

Participants reported that the government had set up the eLab in selected health facilities as a document and record control system. However, the initiative is underutilized for several reasons. Firstly, the provided internet access on mobile phones which is meant to facilitate the electronic transmission of pathological and laboratory medicine results for VL and DBS results is used by staff for other purposes including personal use. Secondly, some clinicians prefer the paper-based system, and thirdly, the eLab platform is considered as not very user-friendly. As two respondents explained.

#### Financial resources

Participants reported the lack of funding from the government to effectively run the laboratory services in districts without cooperating partner support. As one interviewee described:

### Service delivery barriers to absorption of PMTCT testing requirements

#### Specimen collection and transportation

The majority of the health facilities especially in the rural district are not equipped to perform all of the necessary laboratory tests (as shown in Table 5), and this requires referring patient samples to higher-tiered laboratories referred to as Hubs, which are well-equipped to perform certain tests. Respondents highlighted the benefit of this service for transporting samples “*The system works, and we can cater to hard-to-reach facilities even those which have no road network*” (Laboratory Personnel, tertiary Institution). However, they also described how this service was undermined by funding and other resource challenges.

#### Specimen processing and transmitting of results

The participants articulated that adhering to the testing requirements of the new guidelines was a challenge as results are often delayed due to specimen testing machines being out of order at referral laboratories. As explained by one respondent.

The health workers felt the current TAT (turnaround time) of pathological and laboratory medicine results for VL and DBS (2–3 weeks in urban districts and 4 weeks in rural areas) was too long and a source of frustration. As explained by one health worker: “*It makes our work difficult and makes you feel like you are not working especially when the results don’t come back, and you are made to ask your client to make fresh submission of samples. Right now, we receive pathological and laboratory medicine results for PMTCT between 2/4 weeks. There is no quality in that”. (*Nurse, Maternal & Child Health Department, PHC Facility).

Health workers also reported that sometimes they experienced a mismatch between the information they provided on the specimen they sent to a referral lab and the information on the results they received from the lab. Sometimes the results were misplaced. These issues made it difficult to communicate the results to patients.

### Existing strategies to support absorption of the test and treat policy

Participants highlighted the Hub and Spoke approach as a strategy put in place to support the absorption of the new policy. They stated that the majority of the primary healthcare facilities that did not have laboratory infrastructure to perform the basic repertoire of tests sent their PMTCT laboratory specimens to the nearest testing laboratory with an expanded test repertoire; referred to as the Hub (tertiary hospital) and Spoke (referring primary healthcare facilities) approach. The hub and spoke facilities were also connected to the electronic logistic management information system (eLMIS).

Participants described the recruitment of additional health workers as a facilitating factor, which was made possible through the support of cooperating partners. This recruitment of more health workers reduced some of the challenges they faced regarding increasing demand for laboratory testing. *“Our partners have helped us a lot to reduce some of the challenges we face in the department. They have recruited laboratory personnel who have different work schedules*” (Laboratory Personnel, Tertiary Institution).

One existing facilitating strategy was the provision of mobile phones provided by cooperating partners, for use with the eLab system.

A programs manager from a cooperating-partner international organization working in the province described how the strategy of working with the government and cooperating partners was important as it enabled the introduction of innovations to strengthen the specimen-referral system: *“We have lined up joint activities between the government and ourselves. We have a dedicated vehicle for transporting specimens from the hub facilities to reference laboratories, this is also integrated with mentoring, support supervision visits and delivering results to the facilities along the way. The presence of this partnership has improved the timely initiation and has increased patient access to treatment* (Program Officer, Cooperating Partner).

### Proposed strategies from the perspective of participants

Study participants were asked to list strategies they would propose for improving the absorption of PMTCT guidelines within the laboratory system in their province. Their responses are presented in Table 6**.** Health workers were of the view that the laboratory network can be strengthened by introducing innovations such as Data to Care, which uses surveillance and other data on clients who come into contact with the primary healthcare facilities by identifying those who require care or have fallen out of care through collaboration with other departments within the facility. As described by one health worker:

**Table 6:** Proposed strategies

**Resource**
***Infrastructure and supply***
-Expand and build laboratory infrastructure and system to non-hub and spoke facilities
-Availability of logistics should reflect the changes in the policies
-Facilitate timely delivery of laboratory logistics and supplies
***Human Resources***
-Human resource support by cooperating partners
-Incentivizing extra working hours for government workers
-Need for government to absorb human resource employed by cooperating partners when their contracts ended
-During the rollout of new guidelines Laboratory staff needed to be among those prioritized since they fall among frontline staff
-Incentive extra working hours for human personnel especially those working in PCR to meet the demand of test and treat
***Knowledge and Information***
-Replacing the paper-based system of reporting to eLab system
-Investment in information, communication and technology infrastructure (computers and internet to facilitate computerization of all or nearly all types of patient data (eRecords) and platforms for communicating and sharing clinical and other data between patients, providers and among providers)
***Finance***
-Government needed to prioritize districts that relied on government funding especially rural districts that did not have other funding opportunities.
***Service Delivery***
-Investment in an inter and intra laboratory network system between facilities, districts and the province
-Introduce Data to Care- system that uses surveillance and other data to identify persons with HIV possibly in need of HIV medical care, investigate their vital and care status, locate them and link them to care [22]

Other respondents believed that the government needed to invest more in upgrading health facilities with the latest information, communication and technology infrastructure to meet some of the requirements as provided by the guidelines.

Respondents did not highlight any strategies relating to finance. However, they appreciated the existence of cooperating partners who support the HIV program and specifically PMTCT. Participants were also quick to point out that the government needs to prioritize supporting districts that have no access to additional funding resources such as the ones from cooperating partners. As the rural districts were heavily affected by relying on government funding only. As one respondent explained: *“In our urban facility we have enough storage capacity to take in as many samples as possible at the health facilities because our partners have supplied us with extra fridges and support.*

*Unfortunately, as someone who has also worked in rural facilities, this is not the case because rural districts do not have this privilege of having partners to supplement government efforts.” *(Nurse, Maternal & Child Health Department, PHC Facility)

## Discussion

In this study, we collected qualitative data to explore how laboratory services could meet the needs of the current PMTCT guidelines and identify the existing shortfalls. The principal factors impeding laboratory services were inadequate laboratory infrastructure and inconsistent supply chain management of laboratory reagents, supplies, and consumables. Additionally, there is a lack of modern equipment in the province, such as PCR services, which are concentrated in only two urban districts out of ten districts in the province. Another drawback identified is the undefined laboratory networks, which are insufficient and affect the quality of PMTCT provision at the point of care. Human and financial resources have not been adequately increased to meet the growing demand for testing. Previous studies have suggested that reliable laboratory services in limited-resource settings, such as Zambia, are crucial for achieving global HIV/AIDS targets of eliminating Mother-to-Child Transmission of HIV by 2030 [23–25], as well as Sustainable Development Goals. Similar studies conducted elsewhere also indicate that functional laboratory services at each tier of health service provision are essential for the effective implementation of the PMTCT program [17–19].

Findings from this study show that despite the existence of PCR and other PMTCT diagnostic services in the province, there are still several barriers to accessing the services, such as the distance for patient specimens to reach centralised laboratories, and this contributed to longer TAT. These findings are consistent with research done in Kenya, Malawi, Nigeria and Tanzania [26]. Therefore, there is a need for the country to develop, harmonize and standardize protocols to overcome the gaps in access to quality pathological and laboratory medicine services between the rural–urban population.

Studies have demonstrated reasons for point-of-care (POC) test stock-outs, including inadequate/underestimation of supply chain management during implementation [27–29] inaccurate documentation and distribution systems [30], lack of physical space [31], poor commodity management [32], which support these study findings. To address the challenges of pathological and laboratory medicine services, especially in rural areas, Peeling and Ronald (2009) recommended, among other things, reliable supply chain management, provision of physical space in health facilities, effective training of laboratory human resources, and information systems [33]. Web-based information systems, such as the use of eRecords, Data to Care, and eLab, have been suggested to improve patient outcomes, prevent stock-outs, and detect high-consumption and low-consumption clinics for redistribution of tests to prevent test expiration [34]. Investment in information, communication, and technology infrastructure, such as computers and the internet, to facilitate the computerization of patient data and platforms for communicating and sharing clinical and other data between patients and providers, has also been found to improve pathological and laboratory medicine services for use by urban and rural health workers [35]. Therefore, Zambia must invest in information, communication, and technology infrastructure for primary healthcare clinics, alongside existing strategies and ongoing activities such as the National Health Insurance Scheme (NHIMA), provision of equipment to existing health facilities at all levels, building a reliable supply chain, and training and posting more health laboratory staff and other cadres to help achieve Universal Health Coverage (UHC).

Our findings further highlight the role of cooperating partnerships in supporting healthcare-service delivery. Other studies exploring laboratory challenges in scaling up HIV in low-income and middle-income countries suggest that in countries such as India, where government spending on healthcare is low, over 70% of patients are treated in the private sector. Therefore, pathological and laboratory medicine services are provided by private entities [36]. In our study, there was a focus on strong partnerships between the public sector and cooperating partners to overcome the challenges of pathological and laboratory medicine services, especially in rural areas where this partnership is only limited to urban areas. The partnership is critical to strengthen the public service delivery of PMTCT at public sector facilities and to strengthen referral networks between rural and urban health facilities. Furthermore, this study found that cooperating partners fund the sample referral networks in Zambia through USAID implementing partners.

To strengthen the capacity of the existing laboratory system to perform and meet the diagnostic and treatment needs for PMTCT, it is important to develop a reliable laboratory network with a strong specimen referral system. The allocation of laboratory infrastructure should not be seen as reserved for urban settings but must be expanded throughout the entire national health system with a major emphasis on rural areas [37, 38].

Although health workers in this study described feeling overwhelmed due to changes in PMTCT guidelines for laboratory tests, they were quick to appreciate the presence of cooperating partners who recruited additional laboratory personnel. However, this support was limited to urban areas. Participants felt that the government needed to develop a strategy to employ more personnel or incentivize extra working hours for laboratory health personnel working in the PCR laboratory, given the increased workload.

In this study, we found that the long turnaround time (TAT) of 2 to 4 weeks was frustrating for health workers, as it may delay the initiation of ART and increase vertical transmission of HIV [28, 39].

Reliable laboratory services are a critical health system input to the realization of preventing mother-to-child transmission of HIV. Strengthening of laboratories plays a vital role in ensuring early clinical decisions by health workers and appropriate care and treatment which is central to PMTCT. Our findings correspond with other studies that confirm the need for strengthening the capacity of laboratory services within national HIV/AIDS programs to meet the demands and needs of population health [40, 41].

### Strength and limitations

This study has several strengths. First, it is the first study conducted in Zambia that focuses on exploring whether and how laboratory services have been prepared to absorb new testing requirements following PMTCT Test-and-Treat policy changes. This enhances our understanding of provider experiences. Secondly, the findings not only describe challenges but also identify opportunities for interventions across the health system in rural and urban areas, as well as at tertiary levels. Lastly, this study had a large and diverse participant pool, with representation from multiple cadres of the provincial health workforce from three districts.

Study limitations include the limited transferability of findings, as laboratory challenges may be specific to certain locations and times. It should also be noted that the findings from this study represent the perspective of frontline health workers. Additionally, the document review was restricted to the MOH website. However, a strength of this approach is that it is the only source where updated policy documents are shared.

## Conclusion

This study shows that the implementation of a test-and-treat policy without considering health system laboratory capacity severely limits the effectiveness of PMTCT programme implementation. In this study, we have documented areas related to health system inputs and laboratory service delivery that require greater consideration and support to absorb the current and future PMTCT testing requirements.

Scaling up laboratory services, especially in rural areas, increasing coverage, and improving the quality of care is also necessary. Introducing innovations such as investing in renewable energy, data-to-care, and point-of-care technologies should be given higher priority. It is anticipated that the challenges and proposed strategies from this study would apply to other low-resource settings similar to Zambia.

## Supplementary Information

**Additional file 1.** COREQ checklist.

## Data Availability

The data generated and analyzed during the current study are not publicly available as they contain information that could potentially identify the participants, compromising their confidentiality. However, the data is available from the corresponding author upon reasonable request.

## Abbreviations

ANC: Antenatal Car
ART: Antiretroviral Drugs
DBS: Dry Blood Spot
eLMIS: Electronic Logistics Management Information System
eMTCT: Elimination of Mother-to-Child Transmission of HIV
HEI: HIV exposed
HIV: Human Immunodeficiency Virus
IDI: In-depth Interview
MOH: Ministry of Health
PCR: Polymerase Chain Reaction
PHC: Primary Health Care
PMTCT: Prevention of Mother-to-Child Transmission of HIV
TAT: Turnaround Time
TB: Tuberculosis
VL: Viral Load
