# Supplementary material for: Laboratory services in the context of prevention of mother-to-child transmission of HIV testing requirements in Copperbelt Province, Zambia: a qualitative inquiry
Source: BMC Health Serv Res. 2023 Jul 14;23:753. doi: 10.1186/s12913-023-09747-3 (PMC10347716; doi:10.1186/s12913-023-09747-3)
Supplement: Supplementary file 1 — Additional file 1. COREQ checklist. [file 12913_2023_9747_MOESM1_ESM.docx]

**Additional File 3: COREQ checklist**

Consolidated criteria for reporting qualitative studies (COREQ): 32-item checklist

| **No** | **Item** | **Guide questions/description** |  |
| --- | --- | --- | --- |
| **Domain 1: Research team and reflexivity** |  |  |  |
| Personal Characteristics |  |  |  |
| 1. | Interviewer/facilitator | Which author/s conducted the interview or focus group? | Jonathan Mwanza (JM) and two research assistants |
| 2. | Credentials | What were the researcher's credentials? *E.g. PhD, MD* | Jonathan Mwanza BA, MPH,MSc  Research Assistant 1 BA, MPH  Research Assistant 2. BA |
| 3. | Occupation | What was their occupation at the time of the study? | Author PhD Student  Research Assistant 1. Researcher  Research Assistant 2. Researcher |
| 4. | Gender | Was the researcher male or female? | Female (Research Assistant 2), Male (JM, Research Assistant 1) |
| 5. | Experience and training | What experience or training did the researcher have? | JM and Research Assistant 1 and 2 – experienced in both qualitative and quantitative research |
| Relationship with participants |  |  |  |
| 6. | Relationship established | Was a relationship established before the study commencement? | No |
| 7. | Participant knowledge of the interviewer | What did the participants know about the researcher? e*.g. personal goals, reasons for doing the research* | The broad aim of the study (interview study to explore the perceived effect of rapid PMTCT guidelines changes in Zambia and to assess the health system's readiness to absorb the changes), researcher affiliation and academic background |
| 8. | Interviewer characteristics | What characteristics were reported about the interviewer/facilitator? e.g. *Bias, assumptions, reasons and interests in the research topic* | Reasons for and interest in the topic are included in the methods section. |
| **Domain 2: study design** |  |  |  |
| Theoretical framework |  |  |  |
| 9. | Methodological orientation and Theory | What methodological orientation was stated to underpin the study? *e.g. grounded theory, discourse analysis, ethnography, phenomenology, content analysis* | Content and Thematic analysis and Health System Dynamic Framework |
| Participant selection |  |  |  |
| 10. | Sampling | How were participants selected? *e.g. purposive, convenience, consecutive, snowball* | Key informants: purposive |
| 11. | Method of approach | How were participants approached? e*.g. face-to-face, telephone, mail, email* | Face-to-face & Telephone to make an appointment |
| 12. | Sample size | How many participants were in the study? | 26 |
| 13. | Non-participation | How many people refused to participate or dropped out? Reasons? | No refusals or drop-outs |
| Setting |  |  |  |
| 14. | The setting of data collection | Where was the data collected? e*.g. home, clinic, workplace* | Key informants: workplace  Patients: private location |
| 15. | Presence of non-participants | Was anyone else present besides the participants and researchers? | No |
| 16. | Description of sample | What are the important characteristics of the sample? *e.g. demographic data, date* | Page 6 (Individual Interviews) |
| Data collection |  |  |  |
| 17. | Interview guide | Were questions, prompts, and guides provided by the authors? Was it pilot tested? | An interview guide was devised and piloted and modified to suit the study objectives |
| 18. | Repeat interviews | Were repeat interviews carried out? If yes, how many? | No, all patients and key informants were interviewed once only. |
| 19. | Audio/visual recording | Did the research use the audio or visual recording to collect the data? | Interviews were recorded using a digital recording device |
| 20. | Field notes | Were field notes made during and/or after the interview or focus group? | Yes |
| 21. | Duration | What was the duration of the interviews or focus groups? | 30-45 minutes |
| 22. | Data saturation | Was data saturation discussed? | No |
| 23. | Transcripts returned | Were transcripts returned to participants for comment and/or correction? | No |
| **Domain 3: analysis and findings** |  |  |  |
| Data analysis |  |  |  |
| 24. | Number of data coders | How many data coders coded the data? | Two researchers (JM and 2 research assistants) |
| 25. | Description of the coding tree | Did the authors provide a description of the coding tree? | No |
| 26. | Derivation of themes | Were themes identified in advance or derived from the data? | Derived from the data |
| 27. | Software | What software, if applicable, was used to manage the data? | NVivo |
| 28. | Participant checking | Did participants provide feedback on the findings? | No |
| Reporting |  |  |  |
| 29. | Quotations presented | Were participant quotations presented to illustrate the themes / findings? Was each quotation identified? e*.g. participant number* | Yes |
| 30. | Data and findings consistent | Was there consistency between the data presented and the findings? | Yes |
| 31. | Clarity of major themes | Were major themes clearly presented in the findings? | Yes |
| 32. | Clarity of minor themes | Is there a description of diverse cases or discussion of minor themes? | Yes |
